# Pharmacy switch of antipsychotic medications: patient’s perspective

**DOI:** 10.1186/s12991-015-0066-y

**Published:** 2015-09-25

**Authors:** Slawomir Murawiec, Aleksandra Rajewska-Rager, Jerzy Samochowiec, Sylwia Kalinowska, Jacek Kurpisz, Joanna Krzyzanowska, Halina Sienkiewicz-Jarosz, Iwona Kurkowska-Jastrzebska, Agnieszka Samochowiec, Przemyslaw Bienkowski

**Affiliations:** Institute of Psychiatry and Neurology, 9 Sobieskiego St., 02957 Warsaw, Poland; Department of Psychiatry, Medical University, Poznan, Poland; Department of Psychiatry, Pomeranian Medical University, Szczecin, Poland; Department of Clinical Psychology, Institute of Psychology, University of Szczecin, Szczecin, Poland

**Keywords:** Antipsychotic treatment, Pharmacy substitution, Generic drugs, Medication switching, Schizophrenia

## Abstract

**Background and aim:**

Several studies have raised concerns over consequences of brand-to-generic and generic-to-generic pharmacy-generated medication substitutions in psychiatric and non-psychiatric patients. The purpose of this retrospective study was to assess behavioral and emotional responses of patients with schizophrenia to antipsychotic medication substitution performed by pharmacies.

**Methods:**

A group of Polish ambulatory patients with schizophrenia (*n* = 196) chronically treated with antipsychotic medications were asked whether antipsychotic medication substitution had been proposed by a pharmacist in the last 12 months. Ninety-nine patients answering positively were administered more questions addressing the patient’s emotional and behavioral response to the pharmacy proposal.

**Results:**

The most important findings of the present study can be summarized as follows: (1) approximately half of the patients were confronted with a pharmacy proposal to switch their antipsychotic medications in the last 12 months, (2) one quarter of these patients did not accept the pharmacy switch, (3) a substantial proportion of patients (>40 %) did not receive any explanation from a pharmacist offering medication substitution, (4) pharmacy-generated substitution proposals were mainly associated with negative patient attitudes and negative emotional responses, (5) substitution proposals provoked an unscheduled psychiatric visit in approx. 10 % of patients, (6) despite the negative attitudes reported by patients, the pharmacy switch rarely led to treatment discontinuation, but did provoke a change in drug dosing in 7 % of patients accepting the switch.

**Conclusions:**

A pharmacy proposal to switch their antipsychotic medications is a relatively common experience of Polish ambulatory patients with schizophrenia. Pharmacy-generated substitution proposals are mainly associated with negative patient attitudes, but rarely lead to antipsychotic treatment discontinuation in this group of patients.

## Background

Antipsychotic medication non-compliance is thought to be a serious threat to the long-term effectiveness of the pharmacotherapy of schizophrenia. Termination of pharmacotherapy and skipping doses can increase the risk of relapse of psychotic symptoms and re-hospitalization [1–3]. Non-compliance may pose a significant burden to the patient, his/her family, and the health care system. Hence, the identification of factors that increase the risk of non-compliance to antipsychotic medications in patients with schizophrenia is of great practical importance [4].

Several studies have raised concerns over therapeutic and clinical consequences of brand-to-generic and generic-to-generic pharmacy-generated medication substitutions in psychiatric and non-psychiatric patients [5–8]. It has been suggested that the uncontrolled pharmacy substitution may be of particular risk for some patient populations, including transplant recipients and epilepsy patients [6, 7, 9, 10]. From this perspective, surprisingly little is known about attitudes of psychiatric patients towards brand-to-generic and generic-to-generic switches and about consequences of the pharmacy substitution to patient compliance. The consequences of pharmacy substitution in psychiatric patients need to be understood when considering patient attitudes towards antipsychotic treatment and how it may modify clinical and social outcomes [2]. For example, one may hypothesize that suspiciousness and anxiety may make psychotic patients less likely to accept medication conversion. It is possible that psychotic patients will be unwilling to take an unfamiliar medication if a new (generic) drug is perceived as different from the one taken thus far [11, 12]. In a single study on this topic, Roman [13] asked 106 Dutch patients with chronic psychotic disorders about their possible reactions to the proposal of exchange of their original antipsychotic medication with a generic counterpart in an artificial situation mimicking a pharmacy visit. A substantial majority of patients (>70 %) stated that they would be unlikely to take a generic antipsychotic if their pharmacist were to substitute their medication with a generic version. When asked for reasons for their negative attitudes, the patients described perceptions of substitutes (which had never been prescribed or administered) as less reliable, less likely to be effective, less user-friendly and more likely to induce adverse effects as compared to their currently taken medication. Notably, a brief standardized explanation accompanying the hypothetical change significantly increased patients’ willingness to take a new drug. Yet the overall attitude to the medication switch remained negative [13].

The market share of generic medicines in Poland is one of the highest in Europe and exceeds 70 % of the total market volume [14]. A unique feature of the Polish market is the availability of tens of different generic medications for many brand-name drugs. For example, more than 50 generic medications containing olanzapine as an active substance have been registered in Poland until November, 2014 [15]. In the Polish market, the term “generic switch” rarely refers to a situation when a brand-name psychotropic drug is replaced with a generic one. In most cases, one generic medication is replaced with another generic medication (multiple-generic substitution) [7]. Pharmacists are forced to propose a new generic antipsychotic medication to a patient whenever a cheaper alternative is available [14, 16]. Any consultation with his/her psychiatrist is not necessary. Given the above, according to the authors’ experience, it is not uncommon to see a Polish patient with schizophrenia who has been exposed to 4–5 different generic medications containing olanzapine (quetiapine, risperidone) in the last 12 months.

The purpose of this retrospective study was to assess behavioral and emotional responses of patients with schizophrenia towards real-life proposals of pharmacy substitution of their antipsychotic medications. For this aim, ambulatory patients with schizophrenia chronically treated with oral antipsychotic medications were asked whether they had been offered by a pharmacist a substitution of their antipsychotic medication in the last 12 months. Patients answering positively were administered a brief questionnaire containing items addressing different aspects of the situation, including patient’s emotional and behavioral response to the pharmacist’s proposal.

## Methods

### Patients

The study was performed in agreement with the ethical standards of the Declaration of Helsinki. The study protocol was reviewed by the Ethics Committee of the Institute of Psychiatry and Neurology, Warsaw, and the authors were informed that formal approval is not necessary. All study subjects gave their written informed consent to participate after study procedures had been fully explained.

Patients routinely treated for schizophrenia in three psychiatric ambulatory services localized in central-west regions of Poland (Poznan, Szczecin, and Warsaw) were recruited to the study from January to December 2013. Caucasian patients, aged 18–65 years, and prescribed oral second-generation antipsychotic medications (amisulpride, olanzapine, quetiapine, risperidone) in the last year were included to the study. Patients treated chronically with antipsychotic long-acting injections were not included to the study. In each case, the diagnosis of schizophrenia (F20) was confirmed by two board-certified psychiatrists according to the ICD-10 criteria.

Potential participants were excluded if their major diagnosis was different from schizophrenia (e.g., schizoaffective disorder, depressive episode, delusional disorder). Patients were also excluded if they had substance use disorders (other than nicotine dependence) or other serious medical condition, which could make communication with the patient difficult (e.g., stroke, dementia).

Two hundred and twenty patients were screened for the inclusion and exclusion criteria. One hundred and ninety-six patients [the mean (±SD) age 41.0 ± 11.9 years, 45.4 % males] were finally recruited. Olanzapine, quetiapine, and risperidone were the most frequently prescribed antipsychotics in this group (olanzapine 38.2 %, quetiapine 29.0 %, risperidone 25.5 %). Alprazolam and valproate were the most frequently co-prescribed psychotropic medications (for details, see Table 1).

**Table 1:** Psychotropic medications taken by 196 patients recruited to the study

Drug	Patients (%)^a^
Antipsychotic medications	
Amisulpride	17.3
Aripiprazole	10.7
Clozapine	8.1
Olanzapine	38.2
Quetiapine	29.0
Risperidone	25.5
Ziprasidone	3.0
Other psychotropic medications^b^	
Alprazolam	10.2
Valproate	13.7

^a^Some patients were treated with more than one psychotropic medication

^b^Other psychotropic medications taken by more than 10 % of study participants

### Study questionnaire

A preliminary version of study questionnaire was constructed by the authors. Items related to pharmacy substitution were selected on the basis of patients’ reports, clinical experience of the authors, and previous studies [13, 17, 18]. A set of eight items related to the pharmacy substitution was decided by a consensus. The preliminary version of the questionnaire was verified for clarity and precision in a pilot study on a group of 15 patients with schizophrenia recruited by the three ambulatory services.

Clinical and socio-demographic data were gathered on the basis of the interview and/or review of medical records. All the clinicians recruiting patients to the study underwent basic training in the questionnaire administration in line with written instructions provided by major authors (S.M., P.B., A.R-R.).

A final version of the study questionnaire contained two parts. The first part contained inclusion and exclusion criteria, the informed consent form, and basic socio-demographic parameters. The second part contained the items related to the pharmacy switch, which were administered in a form of a structured interview by a treating physician (for details, see Table 2). Items 1 and 2 referred to pharmacists’ behavior. Only in case of a positive answer to the first question were the remaining questions administered. Items 3–8 referred to various aspects of the patient’s response to the proposal from general acceptance to more specific emotional and behavioral responses.

**Table 2:** Items of study questionnaire related to pharmacy switch

Introduction	
The questions refer to your visits in pharmacy over the last 12 months with a prescription for antipsychotic medications received from your doctor	

Questions and explanations to patients	Responses and its analyses
1. Were you offered by a pharmacist an antipsychotic medication named differently from that prescribed by your doctor?	Quantitative analysis of yes/no responses
If yes, please, refer to the most recent or most remembered visit and answer the following questions. If no, please, do not answer the other questions^a^	
2. Did you receive any explanations from a pharmacist proposing the switch of the antipsychotic medication prescribed by your doctor?	Quantitative analysis of yes/no responses
3. Did you accept this proposal?^b^	Quantitative analysis of yes/no responses
4. What was your attitude to this situation?	Quantitative analysis of responses on the Likert-like scale
	Very negative
	Negative
	Neutral
	Positive
	Very positive
5. What was your emotional response to this situation?	Qualitative analysis of patient’s descriptions
6. Did you inform your psychiatrist about this situation?	Quantitative analysis of responses
	No
	Yes, during an unscheduled visit
	Yes, during a scheduled visit
7. Did you notice any subjective changes in drug’s efficacy and/or tolerance after the switch?^c^	Quantitative analysis of yes/no responses
	If “yes”, qualitative analysis of patient’s reports
8. Did you change a dose or frequency of antipsychotic drug administration after the switch?^c^	Quantitative analysis of yes/no responses
	If “yes”, qualitative analysis of patient’s reports

^a^Question 1: 99 of 196 patients answered “yes”, 97 of 196 patients answered “no” and were not asked questions 2–8

^b^Question 3: 75 of 99 patients answered “yes” (“acceptors”), 24 of 99 patients answered “no” (“non-acceptors”)

^c^Questions 7–8: administered only to 75 of 99 patients who accepted the proposal of medication switch (“acceptors”)

Quantitative and qualitative approaches were used to provide a more comprehensive description of patient’s perspective and the clinical applicability. Descriptive statistics were used to analyze the data. Special attention was given to those behaviors of patients that could lead to direct clinical consequences (items 7, 8) or to consequences to the mental health care system (item 6; Table 2).

## Results

### Selection of final study group (item 1)

Ninety-nine (50.5 %) of 196 patients recruited to the study answered positively when asked the question whether they had been offered by a pharmacist, in the last year, an antipsychotic medication named differently from that prescribed by their doctor (see item 1; Table 2). These patients were asked the other questions related to the pharmacy switch (see items 2–8; Table 2) and their answers were analyzed further. Basic clinical and socio-demographic characteristics of the final study group (*n* = 99 patients) are shown in Table 3.

**Table 3:** Baseline characteristics of the final study group, i.e., 99 patients who were confronted with the proposal of pharmacy switch

Women (%)	58.5
Age (years)	43.0 ± 11.7^a^
University degree (%)	26.0
Currently employed (%)	27.2
Living with family (%)	80.8
Married or in stable relationship (%)	39.4
Age at onset of the first episode (years)	27.7 ± 8.4
Number of hospitalizations	6.8 ± 6.8
Taking more than one antipsychotic medication (%)	45.5
>5 years of antipsychotic treatment (%)	82.8
Any other psychotropic medications taken (%)	63.6
Any non-psychotropic medication taken (%)	17.2

^a^Mean ± SD

Ninety-seven (49.5 %) of 196 patients recruited to the study who had not experienced pharmacy-generated proposal of antipsychotic medication switch in the last year, did not answer the other seven questions (items 2–8) and their answers were not included in any other statistical analyses.

### Explanations from pharmacist (item 2)

Fifty-six (56.6 %) of 99 patients qualified to the final study group received some explanations from the pharmacist proposing the switch of the antipsychotic medication (item 2). Forty-three (43.4 %) of 99 patients did not receive any explanations from the pharmacist.

### Accepting medication switch (item 3)

Twenty-four (24.2 %) of 99 patients qualified to the final study group did not accept generic substitution in the pharmacy (“non-acceptors”). Seventy-five (75.8 %) of 99 patients qualified to the final study group accepted generic substitution in the pharmacy (“acceptors”; item 3).

### Attitudes towards proposal of medication switch (item 4)

Attitudes towards the proposal of medication switch (item 4) were reported by 96 of 99 patients qualified to the final study group. Three of 99 patients did not report any attitudes to the proposal.

Eleven subjects declared “very negative”, 31 subjects declared “negative”, 36 subjects declared “neutral”, 12 subjects declared “positive”, and six subjects declared “very positive” attitude to the proposal. In general, our data show that negative and very negative attitudes to the proposal dominated over positive and very positive ones (42/96 vs. 18/96). Table 4 presents the distribution of subjective evaluations of the proposal among 96 patients from the final study group divided into the 74 “acceptors” and 22 “non-acceptors” who reported their attitudes. Notably, subjective evaluations in the non-acceptors were shifted towards negative values with the paucity of neutral and absence of positive evaluations.

**Table 4:** Attitudes towards proposal of pharmacy switch in acceptors and non-acceptors

*n* = 96 patients who reported their attitudes	Attitudes towards proposal of pharmacy switch				
	Very negative	Negative	Neutral	Positive	Very positive
Acceptors n = 74^a^	3	21	32	12	6
Non-acceptors n = 22^b^	8	10	4	0	0

^a^Acceptors: patients who accepted the proposal of antipsychotic medication switch, 74 of 75 acceptors reported their attitudes towards the proposal

^b^Non-acceptors: patients who did not accept the proposal of antipsychotic medication switch, 22 of 24 non-acceptors reported their attitudes towards the proposal

### Emotional responses towards proposal of medication switch (item 5)

Sixty-three of 99 patients self-reported their emotional response to the situation they were confronted with in the pharmacy (item 5). Thirty-six of 99 patients did not report any emotional responses to the situation.

Forty-nine of 75 “acceptors” self-reported their emotional response accompanying the generic substitution in the pharmacy. The self-reports were dominated by descriptions of negative emotions (33 of 49 “acceptors”). The most frequent negative emotions, reported by the 32 “acceptors”, were “anger” and/or “fear”. Sixteen of 49 self-reports of “acceptors” referred to positive emotions associated with the pharmacy substitution. Positive emotions reported by the “acceptors” were mostly associated with a lower price of the new medication (nine patients) and a switchback to a medication that had been previously taken (four patients).

Fourteen of 24 “non-acceptors” self-reported their emotional response accompanying the situation they were confronted with in the pharmacy. The self-reports of “non-acceptors” were limited to reports of negative emotions (“anger”, seven patients; “fear”, seven patients).

### Feedback to psychiatrist (item 6)

Fifty-five (55.5 %) of 99 patients qualified to the final study group did not inform their psychiatrists about the proposal of generic substitution. Eleven (11.1 %) of 99 patients (10/75 “acceptors”, 1/24 “non-acceptors”) informed their psychiatrists about the proposed generic substitution during unscheduled visits provoked by the visit in the pharmacy (item 6). Thirty-three (33.3 %) of 99 patients (26/75 “acceptors”, 7/24 “non-acceptors”) informed their psychiatrists during the next scheduled visit.

### Subjective changes in drug’s efficacy, tolerance, and dosing (items 7, 8)

Analyses of responses to the last two questions (item 7, 8) were restricted to 75 patients who accepted the switch (see item 3, Table 2).

Twenty-four of 75 “acceptors” (32 %) noticed subjective changes in drug’s efficacy and/or tolerance (item 7). Twenty-one of 75 “acceptors” (28 %) reported negative consequences of the generic substitution. Decreased tolerance or worse efficacy was reported by 13 and eight of the “acceptors”, respectively. Three patients (4 %) reported positive consequences of the substitution.

Five out of 75 patients (6.6 %) who had accepted a new medication declared that they changed the dose or frequency of their antipsychotic treatment. None of the patients reported treatment discontinuation after the pharmacy switch (item 8; Table 2).

## Discussion

To the best of our knowledge, this is the first study on attitudes towards and consequences of the pharmacy substitution of antipsychotic medications in chronically treated patients with schizophrenia. Although retrospective in nature, the present study provides some important clues in understanding possible consequences of the pharmacy substitution (brand name-to-generic and generic-to-generic) in patients with schizophrenia. The most important findings of the present study can be summarized as follows: (1) approximately half of Polish ambulatory patients with schizophrenia were confronted with a pharmacy proposal to switch their antipsychotic medications in the last 12 months, (2) one quarter of these patients did not accept the pharmacy switch, (3) a substantial proportion of patients (>40 %) did not receive any explanation from a pharmacist, (4) the proposal of a pharmacy substitution was mainly associated with negative patient attitudes, negative emotional responses, and self-reports of decreased tolerance/worse efficacy of a new medication (in the “acceptors”), (5) a substitution proposal provoked an unscheduled psychiatric visit in approx. 10 % of patients, (6) despite the negative attitudes reported by patients, the pharmacy switch rarely led to treatment discontinuation.

The market share of generic medicines in Poland is one of the highest in Europe [14]. A feature of the Polish market is the availability of tens of different generic medications for many brand-name psychotropic drugs. Interestingly, the brand-name medication containing olanzapine was withdrawn from the Polish market and its manufacturer registered olanzapine under a generic name [15]. A common reimbursement price for a group of interchangeable medications can change many times in a given year, providing stimulus to patients, physicians, and pharmacists to switch a medication whenever a new generic counterpart with a lower price is available [14, 19]. Given the above, it is not surprising that more than 50 % of our patients were confronted with a pharmacy substitution proposal of their antipsychotic medication.

In general, the results of the present study tend to support the results of the previous study on Dutch ambulatory patients with schizophrenia or other psychoses. In the latter study, Roman [13] asked patients with chronic psychotic disorders about their possible reactions to a proposal of switching their original antipsychotic medication to a generic counterpart in an artiicial situation mimicking a visit in the pharmacy. A substantial majority of patients (73 %) stated that they would be unlikely to take a generic antipsychotic if their pharmacist were to substitute their medication with a generic version. For obvious methodological reasons, it is difficult to compare 73 % of patients declaring non-acceptance for the switch in the artificial situation provoked in the psychiatric ambulatory settings with 75 % of patients declaring the acceptance for the pharmacist’s proposal in real-life conditions in the present retrospective study. This issue could be addressed in further prospective real-world studies comparing patients’ declarations with their behavior in the pharmacy.

In the study by Roman [13], the patients expected the generic version of their antipsychotic medication to be less reliable, less likely to be effective, less user-friendly, and more likely to produce adverse effects. In line with the above, in the present study, the predominant attitudes to the pharmacist’s proposal in real-life conditions were negative. Moreover, self-reported emotions that accompanied the situation were also mostly negative and included fear and anger. Although the predominant attitudes and emotional responses to the pharmacist’s proposal were negative, the proposal was accepted by 75 % of our patients. Hence, the data may suggest that an apparent difference exists between the formal acceptance of the proposal and experienced attitudes and emotions. Possible explanations for this contrast may come from the abovementioned features of the market, which can habituate psychiatric patients to different generic names of the same active substance [11] and force them to accept generic substitutions of psychotropic and non-psychotropic medications through market regulations and small financial incentives [14]. In a partial agreement with this interpretation, 12 % of our patients who had accepted the pharmacy substitution reported positive emotions associated with a lower price of a new medication.

In the report by Roman [13], 52 % of psychotic patients stated that they would consult their psychiatrists before accepting the generic version of their antipsychotic medication. Most of these patients reported that they would refrain from using the new medication until having spoken to an expert with knowledge of their individual situation in considering whether accepting the switch would be safe and favorable. In agreement with the above observation, in the present study, 45.5 % of the patients informed their psychiatrists about the proposal of pharmacy substitution. Notably, more than 10 % of patients informed their psychiatrists about this situation during unscheduled visits. When comparing the data from the present and previous study, one should remember that ambulatory visits on demand are not easily available for Polish psychiatric patients.

Apart from differences in names, pill colors, and packages, generic medications may differ in terms of plasma drug concentration–time curves. Notably, generic-to-generic switches may vary by more than ±20 % from each other since the pharmacokinetic properties of each generic drug may differ from the brand-name drug in opposing directions [8, 20]. From this perspective, the most unwanted outcome of pharmacy switch of antipsychotic medication in patients with schizophrenia would be a decrease in drug’s efficacy and/or increase in side effects leading, in extreme cases, to treatment discontinuation. Although a decrease in drug’s efficacy and/or safety was reported by approximately one-third of the patients who had accepted a new drug, <10 % of the patients declared that they had changed the dose or frequency of their antipsychotic treatment. The proportion of unscheduled visits and treatment discontinuations reported by our patients can be viewed from different perspectives. Treatment discontinuation even in a small number of patients may generate relatively high costs for the family and health care system [2, 21]. Further studies are needed to estimate real costs and benefits of generic switch in psychotic patients from medical, social, and economic perspectives [22].

The present study involves some limitations. Test–retest reliability of patients’ responses was not tested. The patients’ reports were based on recollections and thus one may expect some memory bias difficult to control and eliminate in a retrospective study. For example, it is possible that some patients discontinued their treatment for a reason unrelated to the pharmacy switch. Yet another problem is that some individuals with a recent experience of pharmacy switch could discontinue their treatment for a reason related to the switch, but after the end of the study.

Another limitation of our study is the relatively small sample size. Although the participants were recruited in three different mental health centers, the sample cannot be treated as fully representative for Polish patients with schizophrenia. On the other hand, to the best of our knowledge, the present study is the first study of real-life subjective experiences associated with pharmacy substitution of antipsychotic medication in individuals suffering from schizophrenia and as such should be treated as a starting point for further in-depth interdisciplinary analyses. Future prospective studies should recruit not only patients but also their psychiatrists and community pharmacists as health care professionals can play a crucial role in generic drug prescription, perception, and acceptance [23, 24].

## Conclusion

A pharmacy proposal to switch their antipsychotic medications is a relatively common experience of Polish ambulatory patients with schizophrenia. Pharmacy-generated substitution proposals are mainly associated with negative patient attitudes and may provoke unscheduled psychiatric visits in approx. 10 % of patients. The pharmacy switch rarely leads to antipsychotic treatment discontinuation, but may provoke a change in drug dosing in some patients accepting the switch.

Our results, taken together with the previous findings [13], tend to support a model in which the pharmacy switch of antipsychotic medications is coordinated with the psychiatrist treating the patient.
